# Spontaneous Bilateral Intraorbital Hematoma: A Case Report of a Child With Sickle Cell Disease

**DOI:** 10.7759/cureus.89745

**Published:** 2025-08-10

**Authors:** Saad Benchekroun, Rim El Hachimi, Meryem Benchekroun, Asmae Azegouar, Lalla Ouafa Cherkaoui

**Affiliations:** 1 Department A of Ophthalmology, Hospital of Specialties, Mohammed V University, Rabat, MAR

**Keywords:** exophthalmos, orbital decompression, orbital hematoma, sickle cell children, sickle cell disease complications

## Abstract

Sickle cell disease (SCD) is an inherited disorder that affects the shape of red blood cells. Spontaneous orbital hematoma is an uncommon complication of SCD. This complication is serious and can affect the visual prognosis. Imaging (computed tomography scan and especially the magnetic resonance imaging) is crucial to diagnose orbital hematomas; it also helps eliminate other causes of rapidly progressive exophthalmos. The management of orbital hematomas in these patients is medical, but a surgical intervention can be necessary. This case highlights the diagnosis and management of this rare complication.

## Introduction

Sickle cell disease (SCD) is a hereditary hemoglobinopathy characterized by the presence of abnormally shaped red blood cells that can obstruct small blood vessels, leading to ischemia and infarction of various tissues [1]. While common complications include painful crises and organ damage, orbital involvement is rare. Orbital hematoma, particularly in the absence of trauma, is an uncommon but serious manifestation of SCD and may result from infarction of orbital bones during a vaso-occlusive crisis [2]. Such hematomas can lead to the sudden onset of eye bulging (exophthalmos), pain, restricted eye movements, and even vision loss if not promptly diagnosed and managed. We present a rare case of a seven-year-old child with SCD who developed a spontaneous bilateral intraorbital hematoma, highlighting the diagnostic challenges and the need for prompt medical and potentially surgical intervention.

## Case presentation

**Figure 1:**
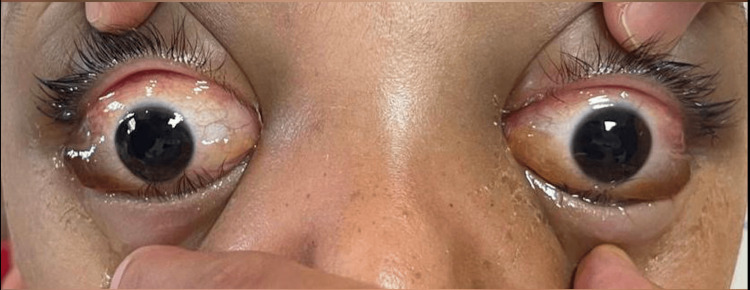
Clinical photograph showing marked bilateral exophthalmos and conjunctival chemosis

We report the case of a seven-year-old boy known to have SCD who was hospitalized in the pediatric unit for a sickle cell crisis with fever and intense pain in the limbs. The patient presented a rapidly progressive bilateral exophthalmos with no history of trauma. The ophthalmological examination found an important bilateral exophthalmos, a total ophthalmoplegia, and a palpebral edema. The eyelid occlusion was impossible due to the severity of exophthalmos. The visual acuity was at counting fingers for the right eye and 4/10 for the left eye, which corresponds to a visual acuity of 2 LogMAR for the right eye and 0.4 LogMAR for the left eye. Both corneas were clear, and the patient had bilateral chemosis. No papilledema was found in the fundus examination. The rest of the ophthalmological examination was normal (Figure 1). The hemoglobin level was at 7 g/dL, and the platelet count was at 275,000/mm^3^.

The orbitocerebral computed tomography (CT) scan showed a bilateral intraorbital hematoma causing bilateral exophthalmos at grade 3 (Video 1).

**Video 1:** Orbitocerebral CT showing a bilateral intraorbital hematoma (yellow arrows) causing bilateral exophthalmos CT: computed tomography

In front of the context and the results of the CT, the diagnosis of a spontaneous bilateral intraorbital hematoma in a sickle cell patient was made. The patient was put on oral corticosteroids in addition to the treatment of the sickle cell crisis that consisted of rehydration, analgesics, blood transfusions, and antibiotics. The following day, no regression of the exophthalmos was noted, and the ophthalmological exam found a visual acuity of counting fingers at 3 m on the left eye with an inferior corneal ulcer due to the exposure of the cornea secondary to the exophthalmos.

**Figure 2:**
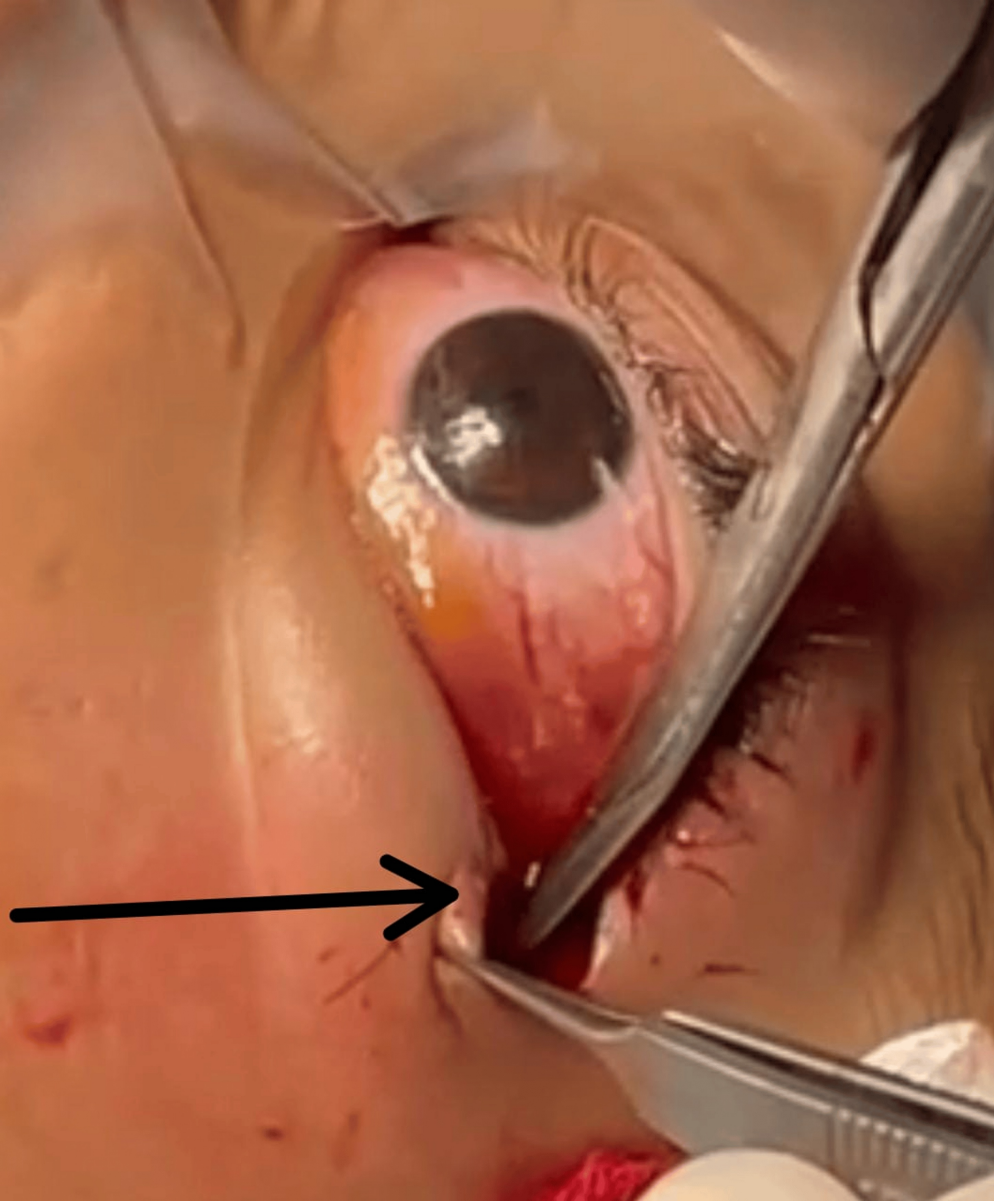
Bilateral lateral canthotomy (black arrow)

Due to the optic nerve compression on the CT, the deterioration of the visual acuity of the left eye, and the corneal complication, we decided to intervene surgically with a bilateral lateral canthotomy and a subperiosteal puncture of the hematoma (Figure 2).

Following the surgery, a decrease in exophthalmos was noted. However, the day after surgery, a recurrence of exophthalmos was noted with a recurrence of the orbital hematoma on CT. Bilateral drainage of the orbital hematoma was performed with immediate regression of exophthalmos (Video 2).

**Video 2:** Bilateral drainage of the orbital hematoma with immediate regression of exophthalmos (green arrow). The yellow arrow shows the blood drained

**Figure 3:**
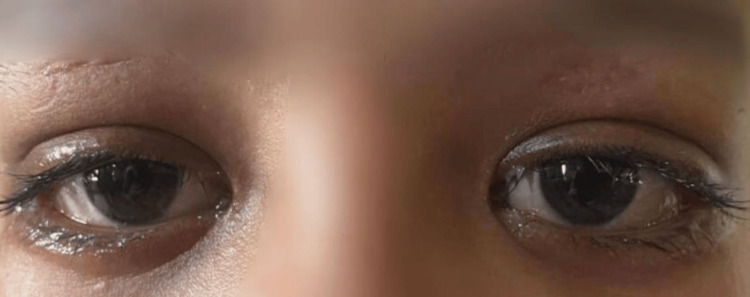
Patient after recuperation of visual acuity and oculomotricity ad integrum

The recuperation of visual acuity and oculomotricity ad integrum was achieved after 10 days, with the total disappearance of the exophthalmos (Figure 3).

## Discussion

SCD represents a group of hemoglobin disorders that are inherited and includes sickle cell anemia [1]. SCD is considered a public health concern in Morocco, with a high prevalence of both the major forms of the disease and the sickle cell trait; 6.5% is the estimated rate of carriers of SCD in Morocco [3]. Orbital hematoma is a relatively rare complication of SCD [2]. The bilateral form of orbital hematoma is rare, too. Indeed, the bilateral form represents only one-third of the described cases of orbital hematoma [4].

The bleeding and formation of the hematoma between the periosteum and the orbital bone are due to the infarction of the orbital bone secondary to a vaso-occlusive process. Indeed, the sickle-shaped red blood cells can lead to the infarction of tissues by blocking small blood vessels [5].

The functional symptoms of orbital hematoma are mainly swelling and ocular pain [3]. Imaging (CT scan and especially the magnetic resonance imaging, MRI) is crucial to diagnose orbital hematomas. It also helps eliminate other causes of rapidly progressive exophthalmos such as orbital rhabdomyosarcoma, orbital cellulitis, orbital extension of retinoblastoma, osteomyelitis, etc. [6].

In most cases, the treatment is medical and includes the management of the sickle cell crisis, including hydration and pain management. Corticosteroids can also be used [3,4,6]. In our case, the medical treatment was not sufficient. Indeed, surgical intervention was necessary due to the compression of the optic nerve, the visual loss, and the corneal ulcer [6].

## Conclusions

Spontaneous bilateral intraorbital hematoma is a rare but potentially vision-threatening complication of SCD, often caused by orbital bone infarction during vaso-occlusive crises. Timely imaging, particularly with CT or MRI, is critical for diagnosis. While most cases can be managed medically, surgical intervention is warranted when there is evidence of optic nerve compression, progressive vision loss, or exposure keratopathy. Clinicians should maintain a high index of suspicion for orbital involvement in sickle cell patients presenting with facial swelling and visual symptoms.
